# Supplementary material for: Histology of the Upper Gastrointestinal Tract, Morphometry and Lymphocyte Subpopulations of the Duodenal Mucosa: Insights from Healthy Individuals
Source: Int J Mol Sci. 2025 Feb 5;26(3):1349. doi: 10.3390/ijms26031349 (PMC11818500; doi:10.3390/ijms26031349)
Supplement: Supplementary file 1 [file ijms-26-01349-s001.zip › Supplementary Material.pdf]

Supplementary Materials:

Supplementary Table S1. Dyspepsia test for healthy volunteers.

Dyspepsia test
**Medical history:**
1. Active smoker (anyone who has smoked any number of cigarettes daily for the past month)<br>☐ Yes ☐ No<br>If yes, please specify the number of cigarettes smoked per day and/or week:<br>2. Alcohol consumption:<br>* A standard drink contains approximately 10 g of alcohol (in Europe). Examples of standard drinks include: 330 ml of 5% beer, 140 ml of 12% wine, 90 ml of fortified wines (e.g., sherry) at 18%, 70 ml of 25% liqueur or aperitif, 40 ml of 40% spirits.<br>* Risky consumption: The World Health Organization (WHO) defines this as the regular daily consumption of 20 to 40 g of alcohol for women, and 40 to 60 g of alcohol for men.<br>☐ Yes ☐ No<br>3. Relevant medical history:<br>☐ Yes ☐ No<br>If the answer is yes, please specify:<br>4. Do you have a 1st or 2nd degree family history of coeliac disease?<br>☐ Yes ☐ No<br>5. Do you have a 1st or 2nd degree family history of inflammatory bowel disease?<br>☐ Yes ☐ No<br>6. Do you have a personal history of neoplasms or inflammatory diseases?<br>☐ Yes ☐ No<br>7. Do you have a history of surgeries?<br>☐ Yes ☐ No<br>If the answer is yes, please specify:
**Usual medication:**
8. Do you take any medication regularly?<br>☐ Yes ☐ No<br>If the answer is yes, please specify:<br>9. Have you used nonsteroidal anti-inflammatory drugs (NSAIDs) in the last 4 weeks?<br>☐ Yes ☐ No<br>If the answer is yes, specify:
**Current clinical status:**
10. Usual bowel habits: Number of bowel movements and their consistency (hard, shaped, soft/pasty, or liquid)?<br>11. Have you experienced any digestive issues that required consultation with a doctor in the last 6 months?

☐ Yes ☐ No

12. Do you experience abdominal distension more than once a month?

☐ Yes ☐ No

13. Do you experience diarrhoea more than once a month?

☐ Yes ☐ No

14. Do you experience constipation more than once a month?

☐ Yes ☐ No

15. Have you experienced bloody diarrhoea in the last 6 months?

☐ Yes ☐ No

16. Have you experienced gastroenteritis in the last 6 months?

☐ Yes ☐ No

17. Do you experience stomach or abdominal pain more than once a month?

☐ Yes ☐ No

18. Have you experienced unintentional weight loss in the last 6 months?

☐ Yes ☐ No

If the answer is yes, please specify:

19. Have you experienced gastrointestinal bleeding at any time in the last 6 months?

☐ Yes ☐ No

20. Have you experienced clinical symptoms such as progressive difficulty or pain in swallowing, heartburn, persistent vomiting, or jaundice (yellowing of the skin) in the last 6 months?

☐ Yes ☐ No

21. Have you experienced unexplained iron deficiency anaemia in the last 6 months?

☐ Yes ☐ No

22. Do you regularly follow a varied Mediterranean diet that includes gluten?

☐ Yes ☐ No

If the answer is negative, please specify:

23. Is there a possibility of pregnancy at the present time?

☐ Yes ☐ No

24. Have you travelled to tropical countries in the last 6 months?

☐ Yes ☐ No

If the answer is negative, please specify:

Supplementary Table S2. Analytical study of healthy volunteers.

Analytical study	
1. Haemoglobin<br>☐ g/dL	10. Glomerular filtration rate<br>☐ mL/min/1.73m^2
2. Platelets<br>☐ x10^9/L	11. Prothrombin Time (PT) and International Normalized Ratio (INR)<br>☐ % ☐
3. Leukocytes<br>☐ x10^9/L	12. IgA-tissue transglutaminase antibodies<br>☐ U/mL
4. Neutrophils<br>☐ %	13. Presence of the HLA-DQ2.5<br>☐ Positive ☐ Negative
5. Alanine aminotransferase (ALT)<br>☐ U/L	14. Presence of the HLA-DQ8<br>☐ Positive ☐ Negative
6. Gamma-glutamyl transferase (GGT)<br>☐ U/L	15. Presence of the HLA-DQ2.2<br>☐ Positive ☐ Negative
7. Alkaline phosphatase (ALP)<br>☐ U/L	16. Presence of the HLA-DQ7.5<br>☐ Positive ☐ Negative
8. Creatinine<br>☐ mg/dL	17. *Helicobacter pylori* serology:<br>☐ Positive ☐ Negative
9. Urea<br>☐ mg/dL	

Supplementary Table S3. Markers, clones, vendors and fluorophores used in this project for the study of intestinal lymphocyte subpopulations by flow cytometry.

Marker	Clone	Vendor	Fluorophore
CD8	SK1	BioLegend, San Diego, CA, USA	BV785
iNKT	6B11 (RUO)	BD Biosciences, San Jose, CA, USA	BV711
CD4	OKT4	BioLegend, San Diego, CA, USA	BV605
LIVE/DEAD		Thermo Fisher Scientific, Waltham, MA, USA	YELLOW
CD3	SK7	BioLegend, San Diego, CA, USA	BV510
CD161	HP-3G10	BioLegend, San Diego, CA, USA	BV421
CD45	5B1	Miltenyi Biotec, Bergisch Gladbach, Germany	PerCP
Vδ2T	B6 (RUO)	BD Biosciences, San Jose, CA, USA	FITC
Vδ1T	REA173	Miltenyi Biotec, Bergisch Gladbach, Germany	PEVio770
CD8β	2ST8.5H7	BD Biosciences, San Jose, CA, USA	PE
CD103	Ber-ACT8	Miltenyi Biotec, Bergisch Gladbach, Germany	APCVio770
TCR Vα7.2	3C10	BioLegend, San Diego, CA, USA	AF700
CD56	REA196	Miltenyi Biotec, Bergisch Gladbach, Germany	APC
CD103	Ber-ACT8	BioLegend, San Diego, CA, USA	BV785
CD335 (NKp46)	9E2	BioLegend, San Diego, CA, USA	BV711
CD161	HP-3H10	BioLegend, San Diego, CA, USA	BV605
CD3	UCHT1	BioLegend, San Diego, CA, USA	BV570
HLA-DR	L243	BioLegend, San Diego, CA, USA	BV510
CRTH2	BM16	BD Biosciences, San Jose, CA, USA	V450
CD14	TuK4	Life Technologies, Carlsbad, CA, USA	FITC
CD303 (BDCA-2)	AC144	Miltenyi Biotec, Bergisch Gladbach, Germanyi	FITC
CD1α	HI149	BioLegend, San Diego, CA, USA	FITC
CD34	581	BioLegend, San Diego, CA, USA	FITC
CD94	DX22	BioLegend, San Diego, CA, USA	FITC
CD123	6H6	BioLegend, San Diego, CA, USA	FITC
FcεRIα	AER-37 (CRA-1)	BioLegend, San Diego, CA, USA	FITC
TCRab	IP26	BioLegend, San Diego, CA, USA	FITC
TCRgd	B1	BioLegend, San Diego, CA, USA	FITC
CD127	R34.34	Beckman Coulter, Brea, CA, USA	R34.34
CD117	104D2D1	Beckman Coulter, Brea, CA, USA	104D2D1
NKp44	Z231	Beckman Coulter, Brea, CA, USA	Z231
CD62L	DREG56	Beckman Coulter, Brea, CA, USA	DREG56
CD56	REA196	Miltenyi Biotec, Bergisch Gladbach, Germanyi	REA196
CD45	2D1	BD Biosciences, San Jose, CA, USA	APC
CD4	OKT4	BioLegend, San Diego, CA, USA	BV510
CD103	Ber-ACT8	BD Biosciences, San Jose, CA, USA	BV421
CD3	SK7	BD Biosciences, San Jose, CA, USA	PERCP
LIVE/DEAD Green		Thermo Fisher Scientific, Waltham, MA, USA	GREEN
TCRαβ	IP26	BioLegend, San Diego, CA, USA	PE-CY7
TCRγδ	11F2	BD Biosciences, San Jose, CA, USA	PE
CD8	RPA-T8	BioLegend, San Diego, CA, USA	APC-H7

CD45	2D1	BD Biosciences, San Jose, CA, USA	APC
CD3	SK7	BD Biosciences, San Jose, CA, USA	PERCP
CD103	Ber-ACT8	BD Biosciences, San Jose, CA, USA	FITC
TCRgd	11F2	BD Biosciences, San Jose, CA, USA	PE
CD45	2D1	BD Biosciences, San Jose, CA, USA	APC
TCRab	WT31	BD Biosciences, San Jose, CA, USA	FITC
